# Selection on different genes with equivalent functions: the convergence story told by *Hox* genes along the evolution of aquatic mammalian lineages

**DOI:** 10.1186/s12862-016-0682-4

**Published:** 2016-05-21

**Authors:** Mariana F. Nery, Brunno Borges, Aline C. Dragalzew, Tiana Kohlsdorf

**Affiliations:** Departamento de Genética, Evolução e Bioagentes, Instituto de Biologia, Universidade Estadual de Campinas, Campinas, Brazil; Departamento de Biologia, FFCLRP, Universidade de São Paulo, Ribeirão Preto, Brazil

**Keywords:** Cetaceans, Pinnipeds, Sirenians, Molecular evolution, *Hox* genes, Positive selection

## Abstract

**Background:**

Convergent evolution has been a challenging topic for decades, being cetaceans, pinnipeds and sirenians textbook examples of three independent origins of equivalent phenotypes. These mammalian lineages acquired similar anatomical features correlated to an aquatic life, and remarkably differ from their terrestrial counterparts. Whether their molecular evolutionary history also involved similar genetic mechanisms underlying such morphological convergence nevertheless remained unknown. To test for the existence of convergent molecular signatures, we studied the molecular evolution of *Hox* genes in these three aquatic mammalian lineages, comparing their patterns to terrestrial mammals. *Hox* genes are transcription factors that play a pivotal role in specifying embryonic regional identity of nearly any bilateral animal, and are recognized major agents for diversification of body plans.

**Results:**

We detected few signatures of positive selection on *Hox* genes across the three aquatic mammalian lineages and verified that purifying selection prevails in these sequences, as expected for pleiotropic genes. Genes found as being positively selected differ across the aquatic mammalian lineages, but we identified a substantial overlap of their developmental functions. Such pattern likely resides on the duplication history of *Hox* genes, which probably provided different possible evolutionary routes for achieving the same phenotypic solution.

**Conclusions:**

Our results indicate that convergence occurred at a functional level of *Hox* genes along three independent origins of aquatic mammals. This conclusion reinforces the idea that different changes in developmental genes may lead to similar phenotypes, probably due to the redundancy provided by the participation of *Hox* paralogous genes in several developmental functions.

**Electronic supplementary material:**

The online version of this article (doi:10.1186/s12862-016-0682-4) contains supplementary material, which is available to authorized users.

## Background

The aquatic mammals – cetaceans (whales, dolphins and porpoises), pinnipeds (sea lions, seals and walruses) and sirenians (manatees and dugongs) – represent three extant mammalian lineages that independently recolonized the aquatic environment. Remarkably, these three groups encompass convergent anatomical and physiological solutions that accommodate the several challenges of aquatic living. Their streamlined body shape minimizes drag, increases performance and reduces transport energetic costs [1]. Drag is also decreased by the presence of either a strikingly reduced pelvic appendicular skeleton or hindlimbs extremely modified and enlarged propulsive appendices [2]; these animals also evolved a pair of paddle-shapes fore-flippers, equivalent to the forelimbs of terrestrial mammals. In all cases, water recolonization involved extensive morphogenetic reorganization and modification of several physiological features [3]. Comparative anatomy and the fossil record have substantially elucidated these evolutionary transitions towards the aquatic life (especially in cetaceans, see [4, 5]). But only recently, the molecular mechanisms underlying such dramatic and convergent morphological changes during evolution of these three mammalian lineages began to be unraveled (e.g. [6–13]).

The convergent evolution of similar traits in response to equivalent selective pressures represents an exceptional opportunity to study which evolutionary processes underlie specific phenotypic modifications associated to the conquest of a new environment. Several recent studies have been dedicated to evaluate the extent to which adaptive phenotypic convergence is attributable to convergent changes at the molecular level [14–17]. Such articles so far demonstrated that convergent evolution of similar phenotypes may or may not share similar molecular bases. Moreover, these similarities may occur at several genetic levels, meaning that the occurrence of convergence may involve the same mutations in the same genes, but may also comprise different and equivalent mutations in the same genes, or even mutations at different genes with equivalent molecular functions [14–17].

Molecular bases engaged in the phenotypic reorganization that occurred during the three evolutionary events of return to the aquatic environment by mammalian lineages likely comprise transcription factors essential for developmental processes. Among these, *Hox* genes are ideal candidates to test for associations between molecular evolution and morphogenetic rearrangements. These transcription factors regulate patterning of specific body structures during development by specifying regional identities along the anterior-posterior axis of all billaterian metazoans [18]. Vertebrates, in particular, possess four distinct *Hox* gene clusters (*Hox* A, B, C and D) located on different chromosomes, as a consequence of the two rounds of whole genome duplications that occurred early in their evolutionary history [19]. Their fundamental role in patterning body morphology, together with their broad contribution to developmental processes (e.g. [20–25]), foster the assertion that variation in nucleotide sequences of *Hox* gene coding regions is usually associated to phenotypic differences [26–31]. As a consequence, this gene family is often recognized as a major agent for diversification of metazoan body plans [19, 20]. Evaluation of molecular evolutionary patterns of *Hox* genes in the context of independent origins of equivalent phenotypes, however, remains relegated [but see [32] for a recent study on the topic].

In this study we test for convergent signatures in *Hox* genes by comparing sequences of three mammalian lineages that are phenotypically very similar: cetaceans, pinnipeds and sirenians. Our goal is to provide a more comprehensive understanding of *Hox* genes molecular evolution in these aquatic lineages that secondarily and independently recolonized the sea, by characterizing and comparing the coding regions of *Hox* genes in aquatic and terrestrial mammals. Specifically, we test whether *Hox* genes underwent different selective pressures in aquatic mammals, in nucleotide and amino acid sequences, and also whether there are molecular signatures of these genes that endorse convergent developmental mechanisms during evolutionary transitions towards re-colonization of aquatic environments.

## Methods

In order to accomplish a wide taxonomic sampling, we obtained unannotated genomic sequences in Ensembl genome browser and in GenBank from the following 19 mammalian species: tasmanian devil (*Sarcophilus harrisii*), microbat (*Myotis lucifugus*), elephant (*Loxodonta africana*), manatee (*Trichechus manatus*), horse (*Equus caballus*), dog (*Canis familiaris*), ferret (*Mustela putorius*), Weddell seal (*Leptonychotes weddellii*), walrus (*Odobesnus rosmarus*), cow (*Bos taurus*), bottlenose dolphin (*Tursiops truncatus*), baiji dolphin (*Lipotes vexilifer*), minke whale (*Balaenoptera acutorostrata*), mouse (*Mus musculus*), rabbit (*Oryctologus cuniculus*), human (*Homo sapiens*), chimpanzee (*Pan troglodytes*), orangutan (*Pongo abelli*) and marmoset (*Callithrix jacchus*). Despite the low genome coverage of some species, the four *Hox* clusters (A, B, C and D) have been well sequenced, and were complete and located within single scaffolds for most species, excepting the Weddell seal, which cluster A was incomplete, cluster B was fragmented in two scaffolds, and clusters C and D were absent, the microbat, which had the Cluster B fragmented in two scaffolds, and the baiji dolphin, which Cluster C remained absent of databases.

Genomic sequences of *Hox* genes from these species were manually annotated based on comparisons with known exon sequences available for humans, using the programs GenScan [33] and Blas2seq 2.2 [34]. To further corroborate the orthologous relationships of each gene included in our analyses, we conducted phylogenetic reconstructions based on maximum likelihood, using the program RAxML [35]. Moreover, in order to maximize the number of orthologs in each analysis, we conducted a search in GenBank for *Hox* genes from other mammalian species. Using this approach, the species composition of each orthologous *Hox* gene varied from 11 up to 38 species. It means that each *Hox* gene had a unique phylogenetic tree, comprising the ortholog specific set of species recovered from sequenced genomes or GenBank. After this verification of orthologous sequences we finally proceeded with the natural selection analyses. All accession numbers and species included in each *Hox* gene analysis are depicted in Additional file 1: Table S1. All nucleotide sequences were aligned using the program PRANK [36], and nucleotide alignments were generated using the amino acid alignments as a template in the software PAL2NAL [37].

**Fig. 1:**
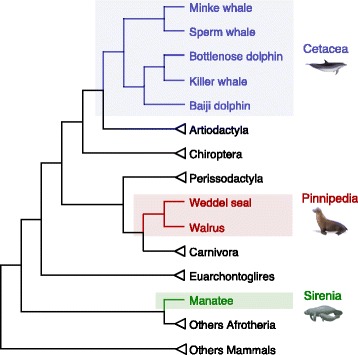
Tree topology used to conduct the analyses of variable ω among lineages; tree assembled from published literature [77, 78]

In order to investigate the possible role of changes in evolutionary rates and to test for positive selection on *Hox* genes evolution in aquatic mammalian lineages, we used the maximum likelihood codon substitution model implemented in the PAML 4.7 package [38]. The following branch-models of variable ω (= *d*_*N*_*/d*_*S*_) rates among lineages were implemented, based on Fig. 1: the *one-ratio model* that assigns the same ω ratio for all branches, and a *two-ratio model*, where one ω was assigned for the ancestral branch of Cetacea, Pinnipedia and Sirenia, treated separately, and another ω was assigned for all remaining mammals. This approach allowed us to identify whether any *Hox* gene in an aquatic lineage exhibits accelerated evolutionary rates when compared to terrestrial counterparts.

We also aimed to detect evidence of positive selection on specific sites along a specific lineage, so we applied branch-site models in PAML, which compare changes in ω along the different sites in the alignment between the foreground branch of primary interest and the background branches [39]. Branches leading to the ancestor of the cetaceans, to the ancestor of pinnipeds, and to the sirenian lineage were chosen as foreground branches in each analysis. We compared the modified model A [40], in which some sites are allowed to change to an ω > 1 in the foreground branch, with the corresponding null hypothesis of neutral evolution. The Bayes Empirical Bayes (BEB) method identified sites under positive selection [40]. In all cases, three starting ω values (0.5, 1.0 and 2.0) were used to verify the existence of multiple local optima. Nested models were compared using the likelihood ratio test (LRT), and the level of significance was settled at 0.05.

Branch-site models may be limiting because they require the prior identification of foreground lineages and the assumption that *d*_*N*_*/d*_*S*_ = 1 for all background lineages [41]. For that reason, we examined each *Hox* gene for signatures of episodic positive selection using a *mixed effects model of evolution* [MEME, 42], performed with HyPhy package implemented in DataMonkey Web Server [43]. The best-fitting nucleotide substitution model was selected through the automatic model tool available on the server, and then ω variation was evaluated among sites and lineages simultaneously. In doing so, this model allows identification of episodes of positive selection that affect only a subset of lineages, which are often ignored using other methods assuming that the ω value is shared by all sites in the alignment or that selective pressures are constant throughout time [42]. In MEME there is no need to specify foreground or background branches. As a complementary approach, we also used another method named BUSTED (Branch-site Unrestricted Statistical Test for Episodic Evolution [44]), implemented in the DataMonkey web server. This method is capable of detecting positive selection that has acted on a subset of branches in a phylogeny at a subset of sites within a gene, and the foreground branch has to be indicated. Sites positively selected were identified at a significance level of P <0.05.

As a final step, we also inferred possible physicochemical differences in *Hox* sequences among the mammalian lineages studied. The aforementioned codon-based analyses implemented in PAML and in DataMonkey do not take into account the magnitude of changes in the physicochemical properties of amino acids resulting from a nonsynonymous substitution. To detect significant physicochemical amino acid changes among residues in *Hox* genes, we used therefore the algorithm implemented in TreeSAAP 3.2 software [45]. This program compares the magnitude of property changes of non-synonymous residues inferred from a phylogeny, and indicates which amino acid properties likely have been affected by positive destabilizing selection during the evolutionary process. In TreeSAAP, the magnitudes of non-synonymous changes are classified into eight categories according to the change in specific physicochemical properties, from conservative (1–3) to very radical substitutions (6–8). For each category, a *z-score* is calculated. Significant positive *z-scores* indicate that a given region is under influence of positive selection (i.e., the number of inferred amino acid replacements significantly exceeds the number of those expected by chance). Here we only considered significant substitutions assigned to the highest categories representing extreme changes in physicochemical properties (categories 7 and 8) at the *P* < 0.001 level. The physicochemical properties identified in these categories were then subjected to a sliding window analysis of 20 codons in width to verify which specific regions in the protein differ significantly from a neutral model.

## Results and discussion

Cetaceans, pinnipeds and sirenians have independently evolved very similar specialized body plans and associated physiological features for an aquatic lifestyle. Molecular mechanisms underlying these key adaptations have recently received increasing interest [9], and the question of whether such convergent phenotypic adaptations share similar molecular bases deserves special attention due to the amount of complete sequenced genomes newly available for several mammals [46, 47]. Similarity may occur at many levels, such as nucleotides, genes, networks and functions [14]. In agreement with the postulate that *Hox* molecular evolution extensively engages major morphological transitions in vertebrates, our results from different analyses focusing on selective regimes acting during *Hox* genes evolution suggest that independent evolution of phenotypically similar aquatic mammalian lineages involved developmental convergence residing on *Hox* functions instead of on *Hox* genes, as further detailed.

Our evaluation of *Hox* genes evolution in aquatic mammals started with annotation of the full complement of structural genes in the *Hox* clusters on unannotated genomic sequences from 19 mammalian species representatives from all major mammalian lineages, an approach where we also included known *Hox* sequences of additional mammalian species retrieved from GenBank. We surveyed all the 39 *Hox* protein sequences, which represent all the gene family members. As expected, synteny is conserved in all species and the cluster size was very similar among lineages (Additional file 2: Table S2). Maximum likelihood phylogenies arranged *Hox* genes into well-supported clades (data not shown), allowing us to establish orthologous relationships of the manually annotated genes.

**Fig. 2:**
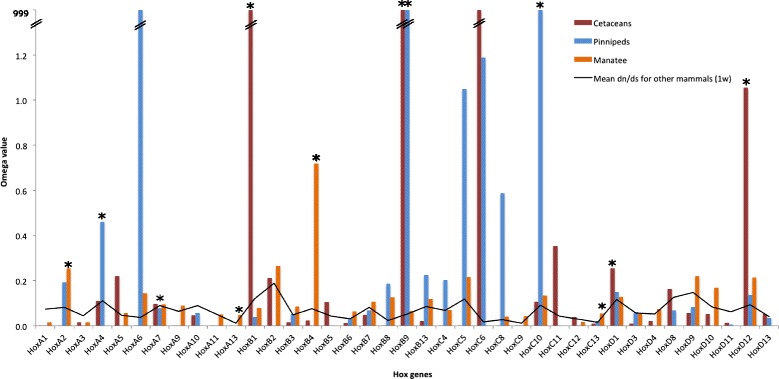
The ω values for *Hox* genes in cetaceans, pinnipeds, sirenian and average value for the remaining mammals (solid line) derived from the two-ratio model. Asterisks indicate those *Hox* genes statistically inferred as evolving under positive selection or those having significantly higher evolutionary rates

We aimed to detect possible roles of positive selection during the evolution of *Hox* genes in aquatic mammalian lineages, so we implemented different models using a maximum likelihood approach in the program PAML and in the program HyPhy within DataMonkey server. As summarized in Fig. 2 (results from the branch models implemented in PAML) and the Additional file 3: Table S3 (likelihood values for all models), several *Hox* genes have evolved under specific selective regimes in one or more mammalian aquatic lineages. The one-ratio model showed that the ω values (ranging from 0.011 in *HoxC9* to 0.189 in *HoxB2*) were significantly lower than 1. These low values were already expected because *Hox* genes, which are known to be pleiotropic, have experienced constrained selective pressures to maintain their function. We then applied the two-ratio model for each *Hox* gene, in order to estimate one ω value separately for Cetacea, Sirenia and Pinnipedia and another ω to all the remaining terrestrial mammals. The LRT tests showed that the two-ratio model fits significantly better than the one-ratio model for *HoxB1*, *HoxB9*, *HoxD1* and *HoxD12* genes in the cetacean lineage, for *HoxA4*, *HoxB9* and *HoxC10* genes in the pinnipedian lineage, and for *HoxA2*, *HoxA13*, *HoxB4* and *HoxC13* genes in the sirenian lineage (Table 1), suggesting that these genes were subjected to different selective pressures during their evolution when compared to other mammalian counterparts. The higher ω values for these genes suggest significantly accelerated rates of evolution in these lineages. Such accelerated rates could either represent relaxation on purifying selection or reflect the action of positive selection. These results suggest that evolution of equivalent phenotypes may have recruited changes in *Hox* genes that were specific in each group.

**Table 1:** Genes and sites inferred to be under positive selection using different models implemented in PAML

Model	Cetacea	Positively selected sites	Pinniped	Positively selected sites	Sirenia	Positively selected sites
Branch model	**HoxB1**, HoxB9, HoxD1, HoxD12	-	HoxA4, HoxB9, HoxC10	-	HoxA2, HoxA13, HoxB4, **HoxC13**	-
Branch-site model	**HoxB1**	18, 35, 64, 147, 153, 169			**HoxC13**	16, 28, 200, 233
MEME	**HoxB1**	64	HoxA7	201	**HoxC13**	233
BUSTED	**HoxB1**	18, 35, 64, 128, 147, 153, 169	HoxA7	201	**HoxC13**	233

In bold are highlighted those genes identified by all methods

Branch models estimate an overall ω value for all codons in the gene, but typically positive selection acts only on a few sites and within a short evolutionary time period [48]. Therefore, we have also explored the variation of evolutionary rates in specific amino acid sites using branch-site models, BUSTED and MEME methods. The branch-site model only identified sites under positive selection in Cetacea (*HoxB1*) and Sirenia (*HoxC13*), while the BUSTED and MEME methods identified episodic positive selection in Cetacea (*HoxB1*), Pinnipedia (*HoxA7*) and Sirenia (*HoxC13*), as shown in Table 1. Differences from the results of each model can be attributed to differences in their underlying assumptions [49]. However, the overlapping results among the three models provides good evidence that these genes were indeed subject to non-neutral selective pressures. For example, both *HoxB1* in cetaceans and *HoxC13* in sirenians were identified as being under positive selection by all models.

**Fig. 3:**
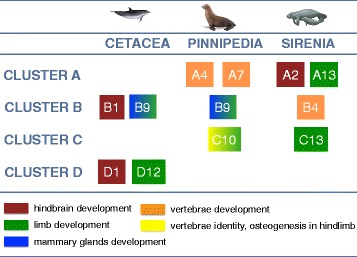
*Hox* genes evolving under positive selection in cetaceans, pinnipeds and sirenians, coupled with their functions

Overall, the signatures of positive selection are much less prevalent across *Hox* genes than those of purifying selection; they are concentrated in few genes and seem exclusive to each aquatic mammalian lineage. The gene *HoxB9* was the only gene found as positively selected in two (cetaceans and pinnipeds) out of the three lineages of aquatic mammals, and none *Hox* gene was inferred as being under positive selection in all three lineages. The apparent lack of a convergence (i.e., different genes being positively selected), however, is revisited when we evaluate the developmental roles of these genes (Fig. 3). The genes *HoxB1*, *HoxD1* (cetaceans) and *HoxA2* (sirenians) are all involved in hindbrain development [50–53]. These are part of the most anterior paralogous groups 1, 2 and 3 that are expressed early during ontogeny and in the anterior axis of the body [54]. Also, the genes *HoxA4*, *HoxA7* (pinnipeds) and *HoxB4* (sirenians) overlap roles related to vertebral development [55–57]. Finally, the genes *HoxB9* (cetaceans and pinnipeds), *HoxD12* (cetaceans), *HoxA13* and *HoxC13* (sirenians) are essential for limb and digit development [58–60]. The gene *HoxC10* was only identified as having accelerated evolutionary rates in pinnipeds; this gene regulates vertebral identity at the transition from thoracic to lumbar and lumbar to sacral regions, and also plays a general role regulating chondrogenesis and osteogenesis in the hindlimb [61]. It is noteworthy that this specific gene had an accelerated evolution rate only in pinnipeds, because this lineage underwent major modifications of their hindlimbs, instead of reduction or loss, which were considerably modified as an adaptation to an amphibian life, since they still use land to reproduce and breed [2].

The substantial overlap among developmental functions of the genes positively selected in aquatic mammals may be interpreted in the light of the history of this complex and unique gene family. It is widely accepted that an ancestral *Hox* gene cluster was duplicated twice during the emergence of vertebrates, as a result of two rounds of whole genome duplications [19, 23]. Consequently, paralogous *Hox* genes, those located at the same relative positions within each of the four clusters, reveal high sequence similarities and shared properties [62]. After duplications, there were secondary losses, and each cluster has selectively retained different subsets of paralogous genes. Those that remained show a strong functional complementarity [63]. The overlapping and complementing functions of the paralogous genes may provide different possible evolutionary routes that achieve the same phenotypic solution for equivalent challenges. In other words, different possible combinations of molecular mechanisms may lead to convergence in trait function, not necessarily through the same molecular solution (i.e. same amino acid substitution in the same gene).

The few studies focusing on *Hox* genes in aquatic mammals relate to cetaceans, and none undergone a comparative analyses of the three aquatic lineages. From these studies, we observed for example that the gene *HoxD12*, previously identified as evolving under positive selection in dolphins [64], was also identified in our analysis as having a significant higher ω value in cetaceans, although we did not identify any particular site evolving under positive selection. This gene is strongly expressed in developing limb buds and is highly conserved across vertebrates [65], but the cetacean sequences are considerably variable when compared to other vertebrates (data not shown here). Also, recent sequence analyses of members from the *Hox* gene family in cetaceans [66] identified significant positive selection only in the gene *HoxB9* in dolphins, at site 175. Our results, just like with *HoxD12*, identified *HoxB9* as having signatures of accelerated evolutionary rates in cetaceans and pinnipeds, but no site was detected as positively selected. As already pointed out by [66], all four paralogous *Hox9* genes act in concert to establish the forelimb posterior domain by regulating *Hand2* expression in this region [67]. Hence, the modified *HoxB9* in cetaceans and pinnipeds might have contributed for acquisition of their fore-flipper. The conflicting results among our analyses and those from previous studies [66, 67] likely reside on the fact that *Hox* genes are subjected to strong long-term purifying selection, which in turn may have hidden any short-term positive selection signal [43]. Therefore, differences in tree topologies and models used for analyses of selective pressures might influence the results. Differently from the other two previous works [66, 67], which used only the dolphin as a cetacean representative and partial coding sequences, here we included complete sequences for all species considered, and always had more than one cetacean representative in our analyses, besides using several models with different assumptions. Such methodological design endorsed accuracy and strictness to our study.

Recently, two independent studies used genomic approaches to investigate the molecular basis of the convergent evolution of cetaceans, pinnipeds and sirenians [46, 47]. The genomic analyses from [46] found that convergent amino acid substitutions were widespread throughout the genome and that a subset of these substitutions was in genes evolving under positive selection. They concluded that convergent evolution likely most arises from different molecular pathways to reach the same phenotypic outcome. Likewise, [47] found convergence in protein coding genes along the whole genome associated with aquatic lifestyle is mainly characterized by independent substitutions and relaxed purifying selection. Both genomic studies [46, 47] did not focus specifically on the *Hox* gene family or assessed the possible functional convergence among genes identified as being positively selected. But in accordance to our results, they propose that sequence convergence in aquatic mammals is predominantly characterized by independent rather than parallel substitutions.

In the past decades, research on molecular mechanisms of convergent phenotypes has expanded greatly, and the results have demonstrated that convergent phenotypic evolution may be attributable to similar molecular bases [14, 15, 17]. Such similarities may however occur at several genetic levels, meaning that phenotypic convergent adaptation might emerge through identical convergent mutations (e.g. [68–70]) but may also comprise numerous alternative pathways (e.g. [71–73]). Regarding such alternative pathways, recent studies even suggest that convergence would be more properly accessed through evaluation of gene functions and functional complexes as selection ultimately targets multigenic functional groups instead of unique amino acid sites [74, 75]. Similarly to our results, these studies demonstrate equivalent biological processes undergoing accelerated evolution in phenotypically similar animals, although through changes in lineage-specific sets of genes [71], adding evidence to the statement that convergent evolution involves a mosaic of molecular changes. Moreover, as pointed out before by [16], expanding our view to the several possible levels of the convergence spectrum has the potential to reveal new insights about the mechanisms underlying phenotypic convergence.

The relevance of *Hox* genes for phenotypic evolution endorses discussion about the functional implications of the amino acid properties that are under selection. Our TreeSAAP analyses, which compare the observed distribution of physicochemical changes inferred from a phylogenetic tree with an expected distribution based on the assumption of completely random amino acid replacement that is expected under the conditions of selective neutrality [45, 76], provide evidence for significant physicochemical amino acid changes among residues in *Hox* genes of aquatic mammalian lineages. Similar to our results of the codon models, negative selection dominates the categories of radical changes, but significant physicochemical amino acid changes were detected in all *Hox* genes identified previously as having sites under positive selection or showing significantly accelerated rates of evolution. Evidence for positive selection was recognized in five physicochemical properties, with global *z-scores* above the significance threshold (z > 3.09). These *Hox* genes exhibited at least one physicochemical property under positive destabilizing selection, which is known to interfere both at chemical and structural levels. The properties were: alpha-helical tendencies (*HoxA2*, *HoxA4*, *HoxA13*, *HoxB1*, *HoxB4*, *HoxC10*, *HoxC13* and *HoxD1*), power to be at the C-terminal (*HoxA4*, *HoxB1*), turn tendencies (*HoxA2*, *HoxB1*, *HoxB4*), coil tendencies (*HoxB1*, *HoxB4*) and thermodynamic transfer hydrophobicity (*HoxD12*) (Table 2, Additional file 4: Figure S1). The positive-destabilizing selection acting on such properties in these genes might contribute for adaptive evolution by influencing the gene biochemical and conformational character.

**Table 2:** Amino acid physicochemical properties under positive destabilizing selection in *Hox* genes

Genes	Alpha-helical tendencies	Turn tendencies	Power to be at the N-terminal	Coil tendencies	Thermodynamic transfer hydrophobicity
*HoxA2*	x	x			
*HoxA4*	x		x		
*HoxA7*					
*HoxA13*	x				
*HoxB1*	x	x	x	x	
*HoxB4*	x	x		x	
*HoxC10*	x				
*HoxC13*	x				
*HoxD1*	x				
*HoxD12*					x

Identifying molecular signatures in the *Hox* gene family in three mammalian lineages that independently recolonized aquatic environments highlights the role of changes in developmental functions during recurrent evolution of similar phenotypes. It is important, however, to note that aquatic adaptations of mammals likely involve both changes on coding genes and on elements regulating expression patterns. Whether changes in *Hox* expression patterns contributed more to morphological adaptations in aquatic mammals than changes in *Hox*-protein-function remains to be answered. We can, though, assure that at least some of the *Hox* members belonging to this important gene family were subjected to positive selection during the evolution of cetaceans, pinnipeds and sirenians, and very likely played an important role in the evolution of aquatic adaptations. More importantly, our study kindles the convergence of *Hox* gene developmental functions as a major factor underlying independent evolution of similar phenotypes in three mammalian aquatic lineages. In this way, our results contribute to the ongoing discussion regarding the molecular basis of convergence, adding information to the growing body of evidence that indicate that convergence is hierarquical and may occur at several biological levels.

## Conclusions

This study provides a detailed characterization of the pattern of selection pressures for the entire *Hox* gene family in mammals, and analyzes the extent of positive selection events on these genes with special focus on three aquatic lineages. All *Hox* genes investigated here experienced strong purifying selection, suggesting a conservative general evolutionary pattern in mammals, and positive selection affects only a specific fraction of sites. However, there is substantially more overlap at the level of their developmental functions than of their nucleotide sequences, reflecting the functional complexity of the *Hox* gene family, which is composed by paralogous groups having complimentary functions. Because of their evolution mode (i.e. four extant clusters originated from two events of duplication of only one ancestral cluster), the *Hox* gene family appears to be relatively ‘loose’ in the sense that distinct lineages exhibit convergent molecular evolution involving similar developmental functions that are not settled on the exact same genes.

### Availability of data and material

The accession numbers from Ensembl and GenBank of the dataset supporting the conclusions of this article are included within Additional file 1: Table S1.

## Additional files

Additional file 1: Table S1. Accession numbers of all mammalian species included in our study and the total number of species included in each *Hox* gene analysis. (XLSX 64 kb) Additional file 2: Table S2.
*Hox* clusters size of those mammals with sequenced genomes included in our study. (DOCX 114 kb) Additional file 3: Table S3. Log likelihhod (lnL), number of parameters (np), omega values (ω) and Likelihood Ratio Test (LRT) values under different models for each *Hox* gene. (DOCX 146 kb) Additional file 4: Figure S1. Sliding window plots of the *z-scores* of radically transitions of amino acid properties showing protein regions under positive destabilizing selection in *Hox* genes. (PDF 394 kb)

## Abbreviations

BEB: Bayes Empirical Bayes
LRT: likelihood ratio test
MEME: mixed effects model of evolution
BUSTED: Branch-site Unrestricted Statistical Test for Episodic Evolution
