# Supplementary material for: Selection on different genes with equivalent functions: the convergence story told by Hox genes along the evolution of aquatic mammalian lineages
Source: BMC Evol Biol. 2016 May 21;16:113. doi: 10.1186/s12862-016-0682-4 (PMC4875654; doi:10.1186/s12862-016-0682-4)
Supplement: Additional file 2: Table S2. — Hox clusters size of those mammals with sequenced genomes included in our study. (DOCX 114 kb) [file 12862_2016_682_MOESM2_ESM.docx]

Supplementary Table 2. Hox clusters size of mammalian species with sequenced genomes considered in our study.

| Species  (common name) | Taxonomic Order | Ensembl Location | | Cluster size (number of bases)* |
| --- | --- | --- | --- | --- |
| **Mammalia**  **Eutheria** | | | | |
| *Homo sapiens*  (human) | Primates | Cluster A | Chromosome 7 | 105,637 |
|  |  | Cluster B | Chromosome 17 | 199,046 |
|  |  | Cluster C | Chromosome 12 | 116,298 |
|  |  | Cluster D | Chromosome 2 | 97,251 |
| *Pan troglodytes*  (chimpanzee) | Primates | Cluster A | Chromosome 7 | 105,650 |
|  |  | Cluster B | Chromosome 17 | 205,367 |
|  |  | Cluster C | Chromosome 12 | 121,686 |
|  |  | Cluster D | Chromosome 2B | 99,392 |
| *Pongo abelli*  (orangutan) | Primates | Cluster A | Chromosome 7 | 107,895 |
|  |  | Cluster B | Chromosome 17 | 221,485 |
|  |  | Cluster C | Chromosome 12 | 117,944 |
|  |  | Cluster D | Chromosome 2B | 99,098 |
| *Callithrix jacchus*  (marmoset) | Primates | Cluster A | Chromosome 8 | 118,408 |
|  |  | Cluster B | Chromosome 5 | 196,511 |
|  |  | Cluster C | Chromosome 9 | 122,402 |
|  |  | Cluster D | Chromosome 6 | 100,448 |
| *Mus musculus*  (mouse) | Rodentia | Cluster A | Chromosome 6 | 104,040 |
|  |  | Cluster B | Chromosome 11 | 172,696 |
|  |  | Cluster C | Chromosome 15 | 114,804 |
|  |  | Cluster D | Chromosome 2 | 96,079 |
| *Oryctolagus cuniculus*  (rabbit) | Lagomorpha | Cluster A | Chromosome 10 | 102,778 |
|  |  | Cluster B | Chromosome 19 | 163,612 |
|  |  | Cluster C | Chromosome 4 | 112,230 |
|  |  | Cluster D | Chromosome 7 | 126,244 |
| *Loxodonta africana*  (elephant) | Proboscidea | Cluster A | Scaffold 5 | 105,044 |
|  |  | Cluster B | Chromosome 31 | 157,551 |
|  |  | Cluster C | Chromosome 2 | 115,378 |
|  |  | Cluster D | Chromosome 3 | 94,898 |
| *Trichechus manatus*  (manatee) | Sirenia | Cluster A  Cluster B  Cluster C  Cluster D | NW_004443986  NW_004443990  NW_004443963  NW_004443973 | 103,558  166,787  114,147  95,727 |
| *Canis familiaris*  (dog) | Carnivora | Cluster A | Chromosome 14 | 106,960 |
|  |  | Cluster B | Chromosome 9 | 168,354 |
|  |  | Cluster C | Chromosome 27 | 113,025 |
|  |  | Cluster D | Chromosome 36 | 94,202 |
| *Mustela putorius*  (ferret) | Carnivora | Cluster A | GL896987 | 106,606 |
|  |  | Cluster B | GL897006 | 168,594 |
|  |  | Cluster C | GL897098 | 116,260 |
|  |  | Cluster D | GL897095 | 96,511 |
| *Leptonychotes weddellii*  (weddell seal) | Carnivora | Cluster A  Cluster B | NW_006383451  NW_006384051  NW_006384160 | Not complete* |
| *Odobesnus rosmarus*  (walrus) | Carnivora | Cluster A  Cluster B  Cluster C  Cluster D | NW_004450331  NW_004450302  NW_004450550  NW_004450672 | 108,539  166,504  116,080  95,865 |
| *Equus caballus*  (horse) | Perissodactyla | Cluster A | Chromosome 4 | 104,533 |
|  |  | Cluster B | Chromosome 11 | 161,256 |
|  |  | Cluster C | Chromosome 6 | 116,292 |
|  |  | Cluster D | Chromosome 18 | 94,107 |
| *Bos taurus*  (cow) | Artiodactyla | Cluster A | Chromosome 4 | 105,228 |
|  |  | Cluster B | Chromosome 19 | 167,962 |
|  |  | Cluster C | Chromosome 5 | 114,768 |
|  |  | Cluster D | Chromosome 2 | 95,928 |
| *Tursiops truncatus*  (bottlenose dolphin) | Cetacea | Cluster A | NW_004198077 | 113,148 |
|  |  | Cluster B | NW_004198508 | 160,481 |
|  |  | Cluster C | NW_004197589 | 118,381 |
|  |  | Cluster D | NW_004198622 | 105,539 |
| *Lipotes vexilifer*  (baiji dolphin) | Cetacea | Cluster A  Cluster B  Cluster D | NW_006792921  NW_006791954  NW_006777439 | 108,633  171,004  99,198 |
| *Balaenoptera acutorostrata*  (minke whale) | Cetacea | Cluster A  Cluster B  Cluster C  Cluster D | NW_006725732  NW_006726298  NW_006726687  NW_006730567 | 110,975  170,184  117,477  101,136 |
| *Myotis lucifugus*  (microbat) | Chiroptera | Cluster A | Scaffold 429819 | 104,931 |
|  |  | Cluster B | Scaffold 430021  Scaffold 432586 |  |
|  |  | Cluster C | Scaffold 429927 | 114,955 |
|  |  | Cluster D | Scaffold 429772 | 95,072 |
| **Mammalia**  **Metatheria** | | | | |
| *Sarcophilus harrisii*  (tasmanian devil) | Marsupialia | Cluster A | Scaffold 861706 | 125,576 |
|  |  | Cluster B | Scaffold 856828 | 207,432 |
|  |  | Cluster C | Scaffold 861729 | 111,008 |
|  |  | Cluster D | Scaffold 849635 | 102,416 |

* only for those complete and not fragmented clusters
