# Supplementary material for: Selection on different genes with equivalent functions: the convergence story told by Hox genes along the evolution of aquatic mammalian lineages
Source: BMC Evol Biol. 2016 May 21;16:113. doi: 10.1186/s12862-016-0682-4 (PMC4875654; doi:10.1186/s12862-016-0682-4)
Supplement: Additional file 3: Table S3. — Log likelihhod (lnL), number of parameters (np), omega values (ω) and Likelihood Ratio Test (LRT) values under different models for each Hox gene. (DOCX 146 kb) [file 12862_2016_682_MOESM3_ESM.docx]

Supplementary Table 3. Log likelihhod (lnL), number of parameters (np), omega values (ω) and Likelihood Ratio Test (LRT) values under different models tested for each *Hox* gene considered in our study. Red values indicate models that significantly fit better our data based on LRT.

| GENE | MODEL | lnL | np | ω | | LRT | P |
| --- | --- | --- | --- | --- | --- | --- | --- |
| ***HoxA1*** | One Model | -5091.44299 | 57 | 0.074 |  |  |  |
|  | Two-model Cetacean | -5090.19194 | 58 | 0.075 | 0.000 | 2.5021 |  |
|  | Two-model Pinniped | -5090.96770 | 58 | 0.075 | 0.000 | 0.9506 |  |
|  | Two-model Manatee | -5089.54023 | 58 | 0.076 | 0.015 | 3.8055 |  |
| ***HoxA2*** | One Model | -4191.30455 | 53 | 0.087 |  |  |  |
|  | Two-model Cetacean | -4190.73590 | 54 | 0.087 | 0.000 | 1.1373 |  |
|  | Two-model Pinniped | -4190.74878 | 54 | 0.085 | 0.193 | 1.1115 |  |
|  | **Two-model Manatee** | **-4188.58189** | **54** | **0.082** | **0.254** | **5.4453** | **0.0196** |
| ***HoxA3*** | One Model | -5487.09422 | 43 | 0.045 |  |  |  |
|  | Two-model Cetacean | -5486.43635 | 44 | 0.046 | 0.016 | 1.3157 |  |
|  | Two-model Pinniped | -5486.71592 | 44 | 0.045 | 0.000 | 0.7566 |  |
|  | Two-model Manatee | -5485.81924 | 44 | 0.046 | 0.016 | 2.5499 |  |
| ***HoxA4*** | One Model | -2804.47980 | 23 | 0.115 |  |  |  |
|  | Two-model Cetacean | -2804.47623 | 24 | 0.116 | 0.111 | 0.0071 |  |
|  | **Two-model Pinniped** | **-2802.51854** | **24** | **0.110** | **0.462** | **3.9225** | **0.0476** |
| ***HoxA5*** | One Model | -2731.88867 | 57 | 0.047 |  |  |  |
|  | Two-model Cetacean | -2731.34779 | 58 | 0.047 | 0.220 | 1.0817 |  |
|  | Two-model Pinniped | -2731.88864 | 58 | 0.047 | 0.000 | 5.6E-05 |  |
|  | Two-model Manatee | -2731.87887 | 58 | 0.047 | 0.055 | 0.0196 |  |
| ***HoxA6*** | One Model | -2723.36838 | 63 | 0.038 |  |  |  |
|  | Two-model Cetacean | -2723.03005 | 64 | 0.038 | 0.000 | 0.6766 |  |
|  | Two-model Pinniped | -2721.55668 | 64 | 0.037 | 999.000 | 3.6233 |  |
|  | Two-model Manatee | -2722.00816 | 64 | 0.036 | 0.144 | 2.7204 |  |
| ***HoxA7*** | One Model | -3949.99954 | 61 | 0.090 |  |  |  |
|  | Two-model Cetacean | -3949.99120 | 62 | 0.090 | 0.097 | 0.0166 |  |
|  | Two-model Pinniped | -3949.98584 | 62 | 0.090 | 0.078 | 0.0273 |  |
|  | Two-model Manatee | -3949.99727 | 62 | 0.090 | 0.096 | 0.0045 |  |
| ***HoxA9*** | One Model | -3591.74585 | 55 | 0.065 |  |  |  |
|  | Two-model Cetacean | -3591.06030 | 56 | 0.065 | 0.000 | 1.3711 |  |
|  | Two-model Pinniped | -3589.89206 | 56 | 0.066 | 0.000 | 3.7075 |  |
|  | Two-model Manatee | -3591.55848 | 56 | 0.064 | 0.090 | 0.3747 |  |
| ***HoxA10*** | One Model | -3337.22042 | 33 | 0.090 |  |  |  |
|  | Two-model Cetacean | -3336.56014 | 34 | 0.092 | 0.046 | 1.3205 |  |
|  | Two-model Pinniped | -3337.21120 | 34 | 0.091 | 0.057 | 0.0180 |  |
| ***HoxA11*** | One Model | -3124.60413 | 49 | 0.051 |  |  |  |
|  | Two-model Cetacean | -3123.60084 | 50 | 0.052 | 0.000 | 2.0065 |  |
|  | Two-model Pinniped | -3123.33551 | 50 | 0.052 | 0.000 | 2.5372 |  |
|  | Two-model Manatee | -3124.60411 | 50 | 0.051 | 0.051 | 5.2E-05 |  |
| ***HoxA13*** | One Model | -1839.69375 | 21 | 0.015 |  |  |  |
|  | Two-model Cetacean | -1839.18682 | 22 | 0.015 | 0.000 | 1.0138 |  |
|  | **Two-model Manatee** | **-1837.55091** | **22** | **0.011** | **0.050** | **4.2856** | **0.0390** |
| ***HoxB1*** | One Model | -6663.35075 | 65 | 0.123 |  |  |  |
|  | **Two-model Cetacean** | **-6654.73254** | **66** | **0.120** | **999.000** | **17.2364** | **0** |
|  | Two-model Pinniped | -6662.53461 | 66 | 0.124 | 0.038 | 1.6322 |  |
|  | Two-model Manatee | -6662.63108 | 66 | 0.125 | 0.079 | 1.4393 |  |
| ***HoxB2*** | One Model | -6488.08405 | 45 | 0.190 |  |  |  |
|  | Two-model Cetacean | -6488.04577 | 46 | 0.189 | 0.213 | 0.0765 |  |
|  | Two-model Manatee | -6487.54760 | 46 | 0.187 | 0.265 | 1.0729 |  |
| ***HoxB3*** | One Model | -4944.37357 | 41 | 0.049 |  |  |  |
|  | Two-model Cetacean | -4943.49074 | 42 | 0.049 | 0.015 | 1.7656 |  |
|  | Two-model Pinniped | -4944.36327 | 42 | 0.049 | 0.053 | 0.0206 |  |
|  | Two-model Manatee | -4943.69523 | 42 | 0.047 | 0.086 | 1.3566 |  |
| ***HoxB4*** | One Model | -2629.57689 | 47 | 0.076 |  |  |  |
|  | Two-model Cetacean | -2628.86122 | 48 | 0.078 | 0.023 | 1.4313 |  |
|  | Two-model Pinniped | -2627.85776 | 48 | 0.078 | 0.000 | 3.4382 |  |
|  | **Two-model Manatee** | **-2627.46131** | **48** | **0.074** | **0.719** | **4.2311** | **0.0396** |
| ***HoxB5*** | One Model | -2879.00906 | 63 | 0.044 |  |  |  |
|  | Two-model Cetacean | -2878.54228 | 64 | 0.043 | 0.105 | 0.9335 |  |
|  | Two-model Pinniped | -2878.82394 | 64 | 0.044 | 0.000 | 0.3702 |  |
|  | Two-model Manatee | -2878.39889 | 64 | 0.044 | 0.000 | 1.2203 |  |
| ***HoxB6*** | One Model | -3257.50645 | 57 | 0.031 |  |  |  |
|  | Two-model Cetacean | -3256.73316 | 58 | 0.032 | 0.012 | 1.5465 |  |
|  | Two-model Pinniped | -3257.50302 | 58 | 0.031 | 0.028 | 0.0068 |  |
|  | Two-model Manatee | -3256.89148 | 58 | 0.030 | 0.064 | 1.2299 |  |
| ***HoxB7*** | One Model | -3134.52102 | 61 | 0.081 |  |  |  |
|  | Two-model Cetacean | -3134.41236 | 62 | 0.082 | 0.049 | 0.2173 |  |
|  | Two-model Pinniped | -3134.51103 | 62 | 0.081 | 0.069 | 0.0199 |  |
|  | Two-model Manatee | -3134.44611 | 62 | 0.081 | 0.107 | 0.1498 |  |
| ***HoxB8*** | One Model | -2196.13080 | 47 | 0.023 |  |  |  |
|  | Two-model Cetacean | -2195.56283 | 48 | 0.023 | 0.000 | 1.1359 |  |
|  | Two-model Pinniped | -2195.17603 | 48 | 0.022 | 0.186 | 1.9095 |  |
|  | Two-model Manatee | -2195.69075 | 48 | 0.022 | 0.127 | 0.8801 |  |
| ***HoxB9*** | One Model | -2940.50822 | 60 | 0.052 |  |  |  |
|  | **Two-model Cetacean** | **-2937.20370** | **61** | **0.052** | **999.000** | **6.6090** | **0.0100** |
|  | **Two-model Pinniped** | **-2937.82930** | **61** | **0.052** | **999.000** | **5.3578** | **0.0200** |
|  | Two-model Manatee | -2940.49471 | 61 | 0.052 | 0.067 | 0.0270 |  |
| ***HoxB13*** | One Model | -5798.15080 | 61 | 0.086 |  |  |  |
|  | Two-model Cetacean | -5796.84116 | 62 | 0.087 | 0.021 | 2.6192 |  |
|  | Two-model Pinniped | -5796.48268 | 62 | 0.084 | 0.226 | 3.3362 |  |
|  | Two-model Manatee | -5797.74762 | 62 | 0.085 | 0.119 | 0.8063 |  |
| ***HoxC4*** | One Model | -1927.709942 | 43 | 0.068 |  |  |  |
|  | Two-model Cetacean | -1927.306871 | 44 | 0.069 | 0.000 | 0.8061 |  |
|  | Two-model Pinniped | -1927.051639 | 44 | 0.065 | 0.203 | 1.3166 |  |
|  | Two-model Manatee | -1927.709876 | 44 | 0.068 | 0.070 | 0.0001 |  |
| ***HoxC5*** | One Model | -2030.240572 | 61 | 0.119 |  |  |  |
|  | Two-model Cetacean | -2029.883585 | 62 | 0.120 | 0.000 | 0.7139 |  |
|  | Two-model Pinniped | -2031.483812 | 62 | 0.119 | 1.050 | -2.4865 |  |
|  | Two-model Manatee | -2029.842971 | 62 | 0.115 | 0.216 | 0.7952 |  |
| ***HoxC6*** | One Model | -1958.314329 | 51 | 0.018 |  |  |  |
|  | **Two-model Cetacean** | **-1955.518627** | **52** | **0.017** | **999.000** | **5.5914** | **0.0180** |
|  | Two-model Pinniped | -1958.314315 | 52 | 0.018 | 1.189 | 2.8E-05 |  |
|  | Two-model Manatee | -1957.991685 | 52 | 0.188 | 0.000 | 0.6452 |  |
| ***HoxC8*** | One Model | -2213.422309 | 65 | 0.026 |  |  |  |
|  | Two-model Cetacean | -2213.135428 | 66 | 0.027 | 0.000 | 0.5737 |  |
|  | Two-model Pinniped | -2213.422326 | 66 | 0.026 | 0.588 | -3.4E-05 |  |
|  | Two-model Manatee | -2213.389727 | 66 | 0.260 | 0.041 | 0.0651 |  |
| ***HoxC9*** | One Model | -2491.495564 | 61 | 0.011 |  |  |  |
|  | Two-model Cetacean | -2491.2938 | 62 | 0.012 | 0.000 | 0.4035 |  |
|  | Two-model Pinniped | -2491.368835 | 62 | 0.011 | 0.000 | 0.2534 |  |
|  | Two-model Manatee | -2490.95535 | 62 | 0.011 | 0.043 | 1.0804 |  |
| ***HoxC10*** | One Model | -3875.459913 | 53 | 0.092 |  |  |  |
|  | Two-model Cetacean | -3875.438845 | 54 | 0.092 | 0.107 | 0.0421 |  |
|  | **Two-model Pinniped** | **-3873.264991** | **54** | **0.091** | **999.000** | **4.3898** | **0.0361** |
|  | Two-model Manatee | -3875.106027 | 54 | 0.091 | 0.134 | 0.7077 |  |
| ***HoxC11*** | One Model | -3504.982156 | 55 | 0.043 |  |  |  |
|  | Two-model Cetacean | -3504.290259 | 56 | 0.042 | 0.355 | 1.3837 |  |
|  | Two-model Pinniped | -3504.787265 | 56 | 0.042 | 0.000 | 0.3897 |  |
| ***HoxC12*** | One Model | -3657.278805 | 59 | 0.029 |  |  |  |
|  | Two-model Cetacean | -3567.245901 | 60 | 0.029 | 0.038 | 180.0658 |  |
|  | Two-model Pinniped | -3657.05227 | 60 | 0.029 | 0.000 | 0.4531 |  |
|  | Two-model Manatee | -3656.9429 | 60 | 0.029 | 0.018 | 0.6718 |  |
| ***HoxC13*** | One Model | -3630.643789 | 43 | 0.017 |  |  |  |
|  | Two-model Cetacean | -3630.37931 | 44 | 0.017 | 0.009 | 0.5289 |  |
|  | Two-model Pinniped | -3630.62266 | 44 | 0.017 | 0.021 | 0.0422 |  |
|  | **Two-model Manatee** | **-3627.905766** | **44** | **0.015** | **0.054** | **5.4760** | **0.0192** |
| ***HoxD1*** | One Model | -5434.211965 | 41 | 0.120 |  |  |  |
|  | **Two-model Cetacean** | **-5432.084348** | **42** | **0.117** | **0.256** | **4.2552** | **0.0391** |
|  | Two-model Pinniped | -5434.174059 | 42 | 0.120 | 0.151 | 0.0758 |  |
|  | Two-model Manatee | -5434.189854 | 42 | 0.120 | 0.128 | 0.0442 |  |
| ***HoxD3*** | One Model | -6118.647356 | 51 | 0.057 |  |  |  |
|  | Two-model Cetacean | -6118.296783 | 52 | 0.056 | 0.009 | 0.7011 |  |
|  | Two-model Pinniped | -6118.64478 | 52 | 0.056 | 0.062 | 0.0051 |  |
|  | Two-model Manatee | -6118.637655 | 52 | 0.566 | 0.052 | 0.0194 |  |
| ***HoxD4*** | One Model | -3423.808945 | 51 | 0.052 |  |  |  |
|  | Two-model Cetacean | -3423.346745 | 52 | 0.053 | 0.021 | 0.9244 |  |
|  | Two-model Pinniped | -3423.661165 | 52 | 0.052 | 0.000 | 0.2955 |  |
|  | Two-model Manatee | -3423.651869 | 52 | 0.051 | 0.073 | 0.3141 |  |
| ***HoxD8*** | One Model | -1932.637224 | 21 | 0.126 |  |  |  |
|  | Two-model Cetacean | -1932.549882 | 22 | 0.123 | 0.164 | 0.1746 |  |
|  | Two-model Pinniped | -1932.284637 | 22 | 0.131 | 0.068 | 0.7051 |  |
| ***HoxD9*** | One Model | -3549.585012 | 35 | 0.148 |  |  |  |
|  | Two-model Cetacean | -3547.90564 | 36 | 0.154 | 0.057 | 3.3587 |  |
|  | Two-model Pinniped | -3549.080081 | 36 | 0.151 | 0.083 | 1.0098 |  |
|  | Two-model Manatee | -3549.246936 | 36 | 0.145 | 0.220 | 0.6761 |  |
| ***HoxD10*** | One Model | -3166.459094 | 73 | 0.083 |  |  |  |
|  | Two-model Cetacean | -3166.357213 | 74 | 0.084 | 0.052 | 0.2037 |  |
|  | Two-model Pinniped | -3165.980895 | 74 | 0.084 | 0.000 | 0.9563 |  |
|  | Two-model Manatee | -3165.850978 | 74 | 0.080 | 0.169 | 1.2162 |  |
| ***HoxD11*** | One Model | -2647.146219 | 23 | 0.063 |  |  |  |
|  | Two-model Cetacean | -2645.554772 | 22 | 0.066 | 0.014 | 3.1828 |  |
|  | Two-model Pinniped | -2644.189777 | 24 | 0.068 | 0.005 | 5.9128 |  |
| ***HoxD12*** | One Model | -5838.398626 | 65 | 0.095 |  |  |  |
|  | **Two-model Cetacean** | **-5834.756535** | **66** | **0.093** | **1.055** | **7.2841** | **0.0069** |
|  | Two-model Pinniped | -5838.357093 | 66 | 0.095 | 0.137 | 0.0830 |  |
|  | Two-model Manatee | -5837.575087 | 66 | 0.094 | 0.214 | 1.6471 |  |
| ***HoxD13*** | One Model | -3583.122533 | 29 | 0.041 |  |  |  |
|  | Two-model Cetacean | -3583.080433 | 30 | 0.041 | 0.052 | 0.0842 |  |
|  | Two-model Pinniped | -3583.110032 | 30 | 0.041 | 0.034 | 0.0250 |  |
