# Supplementary figures and images for: Selection on different genes with equivalent functions: the convergence story told by Hox genes along the evolution of aquatic mammalian lineages
Source: BMC Evol Biol. 2016 May 21;16:113. doi: 10.1186/s12862-016-0682-4 (PMC4875654; doi:10.1186/s12862-016-0682-4)

**HoxA2**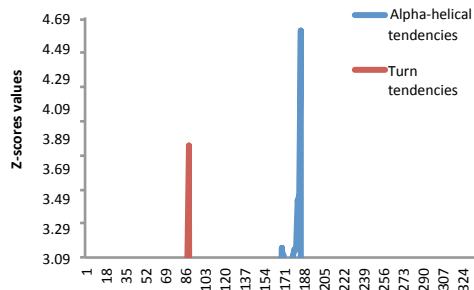**HoxA4**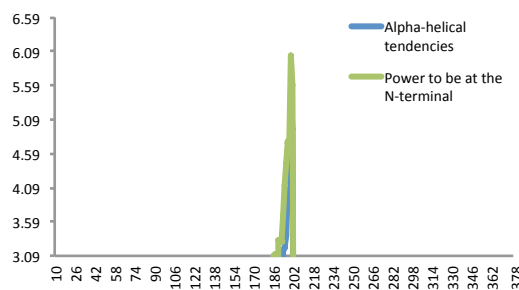**HoxA7**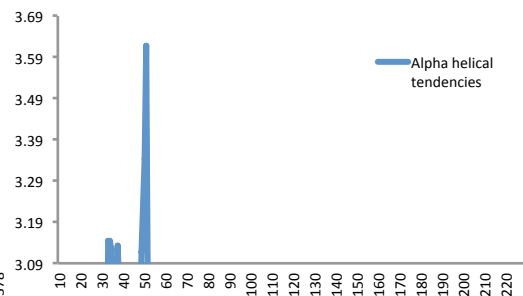**HoxA13**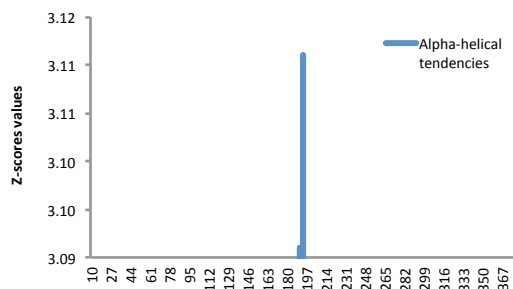

Supplement: Additional file 4: Figure S1. — Sliding window plots of the z-scores of radically transitions of amino acid properties showing protein regions under positive destabilizing selection in Hox genes. (PDF 394 kb) [file 12862_2016_682_MOESM4_ESM.pdf]
